# Longitudinal changes in objectively measured physical activity differ for weekdays and weekends among Chinese children in Hong Kong

**DOI:** 10.1186/s12889-015-2618-0

**Published:** 2015-12-29

**Authors:** Stephen Heung-Sang Wong, Wendy Yajun Huang, Gang He

**Affiliations:** Department of Sports Science and Physical Education, The Chinese University of Hong Kong, Shatin, Hong Kong, China; Department of Physical Education, AAB924, Academic and Administration Building, Hong Kong Baptist University, Kowloon Tong, Hong Kong, China

**Keywords:** Children, Moderate-to-vigorous physical activity, Sedentary behaviour, Longitudinal, Time segments, Accelerometry

## Abstract

**Background:**

Cross-sectional investigation showed that Chinese children in Hong Kong were more physically active on weekends than weekdays, which is contrary to previous findings. However, little is known as to whether these time-segment-specific differences persist with age. This study aimed to compare the 2-year changes in accelerometer-assessed physical activity (PA) and sedentary time (ST) between weekdays and weekends among Chinese children in Hong Kong.

**Methods:**

Children aged 6–8 years were recruited from primary schools in Hong Kong. Time spent in ST (<100 counts per minute [cpm]), moderate-to-vigorous PA (MVPA), and light-intensity PA (LPA) were measured by accelerometer at baseline and then at 1-year and 2-year follow-ups. Mean annual changes were determined using mixed-effects linear models for children who provided 3-day valid data (including 1 weekend day) for at least two time points (*n* = 412). Magnitude of changes between weekdays and weekends was compared using age × time-segment interactions.

**Results:**

At each assessment wave, the percentage of time spent in MVPA (% MVPA) and LPA (% LPA) was consistently high, whereas the percentage of time spent in ST (% ST) was lower on weekends than weekdays. A decrease in % MVPA was found for both weekdays (mean annual change: boys, −0.7, 95 % CI = −0.9 to −0.1; girls, −0.8, 95 % CI = −1.0 to −0.6) and weekends (boys, −1.2, 95 % CI = −1.5 to −0.9; girls, −1.4, 95 % CI = −1.6 to −1.1). An increase was found in % ST for both weekdays (boys, 1.3, 95 % CI = 0.7 to 1.9; girls, 2.4, 95 % CI = 1.9 to 3.3) and weekends (boys, 1.8, 95 % CI = 1.1 to 2.5; girls, 2.6, 95 % CI = 1.9 to 3.3). Mean annual change in MVPA time (min) was greater on weekends than weekdays (difference: boys, 3.0, 95 % CI = 0.3 to 5.7; girls, 3.5, 95 % CI = 1.1 to 5.8).

**Conclusions:**

Age-related decline in MVPA was more marked on weekends than weekdays. Interventions to hinder age-related changes in PA and ST should target both time segments, but weekends warrant particular attention for interventions targeting PA maintenance due to the greater declines.

## Background

Physical activity (PA) is associated with numerous health benefits in children [1]. In addition, excessive sedentary behaviour (particularly when measured as screen-based behaviours) has emerged as a risk factor for cardiometabolic disease independent of moderate-to-vigorous PA (MVPA) [2, 3]. The generally accepted public guidelines recommend at least 60 min per day of MVPA and reducing screen time to less than 2 h a day for school-aged youth [4]. However, many children do not achieve these recommendations, and age-related decline in PA [5] and increase in sedentary time (ST) [6] during childhood and adolescence have been documented in recent systematic reviews. PA declines by 7 % every year during adolescence [5], and objectively assessed daily ST increases by 30 min each year during childhood and adolescence [6]. It was noteworthy that current evidence with regard to longitudinal changes in PA and ST is mainly from western countries.

Many studies have focused on overall PA, but PA levels may vary according to specific time segments of the week. For school-aged children, notable differences in PA levels have been found between weekdays and weekends. A meta-analysis of cross-sectional data showed that children aged 4–18 years spent approximately 14 more minutes per day on PA during weekdays than weekends [7]. The authors anticipated that children have autonomy during weekends and generally do not choose to participate in health-enhancing PA [7]. However, data in this meta-analysis neither accounted for the total time the children wore the accelerometer nor investigated age-related differences. Few studies have compared the magnitude of longitudinal changes in PA and ST between weekdays and weekends, and the findings are equivocal. Brooke et al. [8] reported that for 10-year-old children MVPA declined during both weekdays and weekends over a 4-year follow-up period, but the annual change in MVPA was greater on weekends than weekdays. However, adolescents participating in the PEACH project had relatively stable PA levels from 12 to 15 years of age but accumulated more time in sedentary pursuits during both weekdays and weekends [9]. Furthermore, the magnitude of change in both ST and PA did not differ between school days and weekends [9].

Comparing the magnitude of behavioural change between school days and weekends may be helpful in suggesting intervention strategies to hinder the age-related decline in PA and increase in ST. It may be particularly important for Chinese children for two reasons. First, previous research has suggested weekends to be the promising target for PA maintenance due to the greater age-related declines. Contrary to what has been observed in Caucasian children, a survey conducted in Hong Kong [10] suggests that Chinese youth are more physically active on weekends than weekdays. It remains unknown on whether the difference persists with age. Second, previous studies conducted in Chinese children have been cross-sectional and used subjective measurement of PA [10, 11], which has limited accuracy and precision in estimating the amount and intensity of PA [12]. Therefore, the purposes of this study were to describe the 2-year changes in accelerometer-assessed PA and ST among Chinese children and to examine if the magnitude of the changes differed between weekdays and weekends.

## Methods

### Study design and participants

Data were from the Understanding Children’s Activity and Nutrition (UCAN) study, which was a 3-year longitudinal study investigating determinants of PA and sedentary behaviour in Chinese children in Hong Kong. Ethical approval for this study was obtained from the Survey and Behavioural Research Ethics Committee of the Chinese University of Hong Kong. Primary schools located in different districts were purposely sampled between June and August 2009 to represent a variety of socio-economic status (SES). Among the total of 18 districts, 3 districts with low, medium, or high SES (according to the median domestic household income) were randomly selected. Within each of the selected district, invitations were sent to half of the primary schools. Initially, 27 schools agreed to participate in the study, but 3 of them later dropped out for school-related reasons leaving 24 schools (5 from high SES, 6 from medium SES and 13 from low SES) included (24 % of invited). Written parental consent forms were sent to 6 randomly selected classes in grades 1 to 3 from each participating school. Finally, parental consent was sought from 1666 grades 1–3 children from 24 schools (42 % response rate). The parents of all the participating children were asked to complete a questionnaire for both determinants and PA outcomes [13, 14], and only a subsample of them agreed to their child wearing an accelerometer. The limited number of ActiGraph accelerometers (*n* = 40) that were available for use at the beginning of the study meant that accelerometer data were only collected at baseline from 448 children (26.9 % of the whole sample and 43.8 % of those whose parents had agreed), but at the 1-year and 2-year follow-ups data were collected for all the children whose parents had agreed. The details of numbers at each time point are shown in Table 1. No differences were found in sex or body mass index (BMI) between those who wore the accelerometers and those who did not. However, the children who wore the accelerometers were older and had parents of lower educational attainment than those who did not wear the accelerometer. The current analysis was restricted to accelerometer-assessed activity outcomes.

**Table 1:** Characteristics of the sample at the three time points

	Baseline	1-yr follow-up	2-yr follow-up	≥2 time points^a^
N consented to wear ActiGraph/distributed	1023/448	874/874	706/706	NA
N with returned ActiGraph	445	852	693	NA
N with ≥3 days of PA data including 1 weekend day (% of distributed sample)	263 (58.7)	537 (61.4)	421 (59.6)	412
Age (yrs)	7.8 (1.0)	8.6 (1.0)	9.5 (1.0)	7.6 (1.0)
Gender (% boys)	52.6	52.8	54.8	54.0
BMI (kg⋅m^−2^)	17.3 (3.1)	17.7 (3.3)	18.2 (3.5)	17.1 (3.0)
Overweight and obese (%)	25.8	24.6	26.2	24.1
Parental education level	4.3 (1.4)	4.5 (1.5)	4.5 (1.5)	4.5 (1.5)
Having sibling (%)	62.7	64.9	65.2	66.1
Marital status (% single parent)	12.9	10.4	10.4	8.8
Wearing time (min⋅d^−1^)	789 (90)	796 (78)	801 (90)	793 (86)
MVPA (min⋅d^−1^)	61.5 (23.3)	50.0 (20.4)	45.6 (20.7)	64.2 (22.7)
LPA (min⋅d^−1^)	323.2 (67.3)	329.6 (62.4)	314.8 (64.4)	326.5 (65.8)
ST (min⋅d^−1^)	402.1 (73.2)	414.7 (72.3)	439.8 (80.1)	402.9 (72.5)
% MVPA	8.2 (3.1)	6.4 (2.6)	5.9 (2.8)	8.4 (3.0)
% LPA	40.8 (6.6)	41.0 (6.4)	38.8 (6.9)	40.8 (6.5)
% ST	51.0 (8.1)	52.5 (7.6)	55.2 (8.3)	50.7 (7.9)

Values are means (SD) unless otherwise specified; *MVPA* moderate-to-vigorous physical activity; *LPA*, light-intensity physical activity; *ST* sedentary time; *BMI* body mass index

^a^For those who provided valid accelerometer data for at least two time points (*N* = 412), baseline data are presented

Baseline data (T1) were collected in two school semesters (September to November 2009 and January to April 2010). To minimise seasonal variations, follow-up measurements 1 year (T2) and 2 years (T3) later were conducted as close to the baseline time of year as practicable. Anthropometric data were collected during school visits by trained assessors. Meanwhile, ActiGraph accelerometers and questionnaires were distributed to the children and their parents.

### Measurement of physical activity and sedentary time

The children were each instructed to wear an ActiGraph GT3X accelerometer (ActiGraph, Pensacola, Florida, USA), which was attached to an elasticised belt worn at hip level for 8 consecutive days. The accelerometer was only removed during swimming, showering and sleeping. A 1-min epoch was set to record data. Three days of accelerometer data (including 1 weekend day) with a minimum of 10 h’ recording per day were considered to be valid [8, 15]. Accelerometer data were downloaded using ActiLife 6, and screened and analysed using MeterPlus software (Santech Inc., V.4.3, http://www.meterplussoftware.com). Age-specific cut-off counts [16] were applied to quantify time spent in MVPA (≥4 METs; a 4 MET intensity represents 4 times resting energy expenditure) [17] and light-intensity PA (LPA). For example, the cut-off point for 4 METs corresponded to 1910 cpm for 10-year-old children. A threshold of <100 cpm was used to define ST [18]. After accounting for total wearing time at different time points, three constructs were generated: MVPA, expressed as a percentage of daily wearing time spent in MVPA (% MVPA); LPA, expressed as a percentage of daily wearing time spent in LPA (% LPA) and ST, expressed as a percentage of daily wearing time spent in sedentary behaviours (% ST). Daily minutes in different intensities of PA and ST were also calculated. These outcome measures were derived for the whole week and separately for weekdays and weekends. Cross-sectional comparisons between weekdays and weekends were conducted for children who provided valid accelerometer data for at least 2 weekdays and 1 weekend day at each time point (*n* = 263 at T1, *n* = 537 at T2, and *n* = 421 at T3). Overall, 412 children (222 boys) with valid accelerometer data from at least two time points were included in the analysis to compare the mean annual changes for weekdays and weekends.

### Anthropometric and socio-demographic factors

The children were weighed in the minimum clothing possible and they were measured for height while standing without shoes. Their BMIs were subsequently calculated by dividing their weight (kg) by their height squared (m^2^). Overweight and obesity were classified according to the international standard for children using age- and sex-specific cut-off points [19]. The demographic information was reported by parents, including the responding parent’s age, sex, educational attainment, marital status, the number of siblings, and the child’s sex and date of birth.

### Statistical analyses

The differences in baseline socio-demographic characteristics between the children with valid data from at least two time points and the remaining baseline sample were tested using *t* tests. The participants’ characteristics were also compared at different time points. Mean values (standard deviations) were calculated for the outcome measures because they were approximately normally distributed.

The cross-sectional comparisons at each time point between weekdays and weekends were determined using paired *t* tests. The mean annual changes in the outcomes were determined using linear mixed models controlling for time-vary BMI, parental educational attainment, marital status, sibling numbers and wearing time (for absolute minutes). To account for the clustering by school, a random intercept for the variable ‘school’ was included in the models. Each outcome (MVPA, LPA and ST) was modelled separately. The gender differences in all of the outcomes and gender-age interactions were found in LPA and ST, and the mixed models were therefore separated by gender for the outcomes. Weekday-weekend comparisons in mean annual change were determined by using an age x time-segment interaction term. The statistical analyses were performed using SPSS 22.0 and a probability level of 0.05 was used.

## Results

The socio-demographic characteristics of the sample at different time points were compared and no differences were found in parental educational level, number of siblings or marital status (Table 1). Compared with the baseline sample, the children who provided valid data for at least two time points were more likely to have siblings and less likely to have single parents. However, no differences were found in activity outcomes between these groups.

Table 2 shows the cross-sectional comparisons of MVPA, LPA and ST between weekdays and weekends. At each assessment wave, wearing times were longer on weekdays than weekends. Whereas % MVPA and % LPA were higher on weekends than weekdays, % ST was lower. With regard to absolute MVPA minutes, time-segment differences were only found at baseline (weekdays vs weekends: 61 ± 24 min/day vs 70 ± 37 min/day, *p* < 0.05), not at T2 or T3.

**Table 2:** Cross-sectional comparisons of MVPA, LPA and ST between weekdays and weekends at three time points

	T1 (*n* = 263)		T2 (*n* = 537)		T3 (*n* = 421)	
	Weekday	Weekend	Weekday	Weekend	Weekday	Weekend
Wearing time (min⋅d^−1^)	816 (90)	738 (132)	828 (84)	726 (98)	834 (96)	732 (126)
Daily time in minutes						
MVPA (min⋅d^−1^)	61 (24)	70 (37)	51 (21)	53 (34)	46 (21)	48 (33)
LPA (min⋅d^−1^)	329 (74)	307 (76)	336 (68)	311 (75)	322 (70)	297 (79)
ST (min⋅d^−1^)	420 (79)	356 (108)	436 (79)	360 (91)	463 (86)	380 (107)
Proportion of time (% of wearing time)						
% MVPA	7.6 (2.9)	9.7 (5.3)	6.2 (2.6)	7.3 (4.6)	5.6 (2.5)	6.7 (4.8)
% LPA	40.4 (7.0)	42.0 (7.8)	40.8 (6.7)	43.0 (8.2)	38.7 (6.9)	41.0 (8.8)
% ST	52.0 (8.0)	48.3 (10.5)	53.0 (8.0)	49.7 (10.2)	55.7 (7.9)	52.3 (11.2)

Values are means (SD); MVPA, moderate-to-vigorous physical activity; *LPA* light-intensity physical activity; *ST* sedentary time

All differences between weekdays and weekend days are significant (*p* < 0.05) except for MVPA (min⋅d^−1^) at T2 and T3

No gender-outcome interactions were found; the results were combined for both boys and girls

The comparative mean annual changes in MVPA, LPA and ST are shown in Table 3. Whereas % MVPA and % LPA declined on both weekdays and weekends, % ST increased. The daily declines in MVPA minutes ranged from 6 min for weekdays in boys to 10 min for weekends in girls. The mean annual increases in ST were 19 min/day in girls and 11–13 min/day in boys. The magnitude of annual change in %MVPA was greater on weekends than weekdays for both boys (*B* = 0.5, 95 % CI = 0.1 to 0.9) and girls (*B* = 0.6, 95 % CI = 0.2 to 0.9). No differences in magnitude of change were found in LPA and ST.

**Table 3:** Mean and comparative annual change in MVPA, LPA and ST at weekdays and weekends

	Weekday^a^	Weekend^a^	Difference in annual change^b^ (reference: weekend)
Boys			
MVPA (min⋅d^−1^)	−5.5 (−7.3, −3.6)	−8.5 (−10.6, −6.3)	**3.0** (**0.3**, **5.7**)
LPA (min⋅d^−1^)	−5.8 (−9.4, −2.2)	−4.2 (−8.6, −0.1)	−1.5 (−7.0, 3.9)
ST (min⋅d^−1^)	11.2 (6.8, 15.5)	12.7 (7.4, 18.0)	−1.5 (−8.1, 5.1)
% MVPA	−0.7 (−0.9, −0.1)	−1.2 (−1.5, −0.9)	**0.5** (**0.1**, **0.9**)
% LPA	−0.7 (−1.1, −0.2)	−0.6 (−1.2, −0.0)	−0.0 (−0.8, 0.7)
% ST	1.3 (0.7, 1.9)	1.8 (1.1, 2.5)	−0.5 (−1.3, 0.4)
Girls			
MVPA (min⋅d^−1^)	−6.5 (−8.1, −4.9)	−10.0 (−11.9, −8.1)	**3.5** (**1.1**, **5.8**)
LPA (min⋅d^−1^)	−12.8 (−16.6, −8.9)	−9.3 (−13.9, −4.8)	−3.5 (−9.2, 2.3)
ST (min⋅d^−1^)	19.3 (14.8, 23.8)	19.3 (14.0, 24.6)	−0.0 (−6.6, 6.7)
% MVPA	−0.8 (−1.0, −0.6)	−1.4 (−1.6, −1.1)	**0.6** (**0.2**, **0.9**)
% LPA	−1.6 (−2.1, −1.1)	−1.2 (−1.8, −0.6)	−0.4 (−1.1, 0.4)
% ST	2.4 (1.9, 3.3)	2.6 (1.9, 3.3)	−0.2 (−1.0, 0.7)

Data are presented in β coefficients and 95 % confidence intervals

*MVPA* moderate-to-vigorous physical activity; *LPA* light-intensity physical activity; *ST* sedentary time

^a^Results from linear mixed models for children with valid data for at least 3 days including 1 weekend day ≥2 time points (*n* = 412); β coefficients for weekdays and weekends indicate mean annual change

^b^Comparative annual change between weekday and weekend determined by linear mixed models. β coefficients represent the difference in change between weekday and weekend for every 1-year increase in age. The significant results are in bold. Models were adjusted for time-varying BMI, parental education, marital status, number of siblings, and wearing time (for absolute minutes)

## Discussion

This study examined longitudinal changes of objectively assessed PA and ST over a 2-year period among Chinese children in Hong Kong. Consistent to the findings from the cross-sectional survey in Hong Kong [10], Chinese children accumulated more MVPA time and spent less time in ST on weekends than weekdays and this pattern persisted for two consecutive years. MVPA and LPA declined, and ST increased, on both weekdays and weekends over a 2-year period. Greater declines for MVPA were observed on weekends compared with weekdays. These findings suggest that both weekdays and weekends need to be targeted for interventions to minimise age-related changes in PA and ST for Chinese children. Weekends may be particular important for hindering age-related decline.

This is the first longitudinal study to examine changes in time-segment-specific PA and ST in Chinese children in Hong Kong. The findings on the differences between weekdays and weekends were consistent with the cross-sectional survey in Hong Kong showing that young people seemed to be more physically active on weekends than weekdays [10]. However, opposite results have been found in other countries [8, 20, 21]. As children have more discretionary time during weekends than school days, it is anticipated that they are more likely to choose sedentary activities rather than PA on weekends. A recent systematic review and meta-analysis [7] showed a consistent trend of higher MVPA on weekends than weekdays for school-aged children and adolescents. The authors assumed that the length of weekday and weekend time segments, i.e. the total wearing time of the accelerometer, was comparable [7], but this was not the case for the Chinese children. Children in Hong Kong usually sleep longer and get up later on weekends [22], so the participants had a shorter time to wear the accelerometer. Interestingly, they managed to accumulate more MVPA time despite the shorter wake period on weekends. The results indicated that Chinese children may experience various barriers to participating in PA on weekdays. Recess, physical education (PE) classes, lunchtime and after-school periods are potential PA time periods for children. Although PE is required for school-aged children in Hong Kong, the actual lesson length is 30 % shorter than scheduled and less than half the time is spent in MVPA [23]. In addition, study-related activities, such as homework, occupy most Chinese children’s after-school time [24]. For safety reasons, many schools do not allow their students to play outdoors during recess and lunchtime. This may explain why children spend less time in MVPA and more time in sedentary activities during weekdays.

The age-related decline in PA and increase in ST in Chinese children are in line with previous research [25–28]. Both intensity levels of PA were replaced by ST over the 2-year period. The magnitude of changes was even greater than that previously reported for older children and adolescents. Corder et al. [28] reported an annual increase of 10.6 min/day in ST and a parallel reduction of 3 min/day in MVPA and 9.8 min/day in LPA among 9–10-year-old children. During adolescence, MVPA tends to remain relatively stable, but ST continues to increase at the expense of a reduction in LPA [9]. Children in the current study were in an early primary school year and on average they met the PA recommendations of 60 min/day at baseline. At follow-ups, their mean daily MVPA dropped below the recommendations. Survey studies in Hong Kong have shown that fewer than 50 % of grades 4–6 children achieve the PA recommendations [11, 29] and the prevalence is even lower for adolescents [10]. Such behavioural changes in early childhood may have adverse effects on cardiometabolic health. According to a meta-analysis of multiple cohort studies [30], a 10-min increase in MVPA per day is associated with lower systolic blood pressure, fasting insulin and triglycerides, whereas a 60-min increase in ST leads to higher fasting insulin. Furthermore, the combined association of MVPA and ST with metabolic factors indicated that a difference of 20 min/day in MVPA was associated with 5.6 more cm in waist circumference in children and adolescents [30]. In addition, recent studies have shown that LPA is also important for health. Kwon at al. [31] found that accelerometer-assessed LPA was negatively associated with body fat mass measured by dual energy X-ray absorptiometry among older children aged 8 and 11 years while controlling for MVPA. These findings indicate the urgency of interventions focusing on PA maintenance implemented at an early stage and targeting all intensity levels of PA and sedentary activities.

A difference in magnitude of change between weekdays and weekends was only found in MVPA, not in ST or LPA. Not many longitudinal studies have compared time-segment-specific changes in PA or ST. A greater reduction in MVPA on weekends than weekdays is consistent with the study by Brooke et al. [8], which showed that both total PA (in accelerometer cpm) and MVPA minutes declined to a greater extent on weekends compared with weekdays among British children aged 10 years at baseline. However, ST was not examined in Brooke et al.’s study. Furthermore, British children were more physically active during weekdays than weekends. Identifying the most promising time periods during which to change children’s activity behaviours is believed to be useful for informing future interventions. The lower levels of PA [7] and greater reduction in MVPA on weekends [8] have led some researchers to suggest that the weekend may be a promising time period for interventions to minimise age-related changes in ST and PA. It is noteworthy that although the reduction in PA was substantial at weekends, the Chinese children still managed to accumulate similar or even more time in MVPA within a shorter wake period compared with weekdays at each time point. A better understanding of the factors influencing PA on weekdays and weekends may be helpful to explain this phenomenon. It has been suggested that predictors of 1-year change in PA were different for weekdays and weekends [32]. Parent support was associated with less decline in weekend PA whereas peer support was important in maintaining weekday PA [32]. Both parent and peer support have been found to be associated with PA and screen-based behaviours in children in Hong Kong [29]. Whether the relationships differ between weekdays and weekends need further investigation. In addition, children’s participation in PA is more likely to be influenced by the neighbourhood environment that is unique to Hong Kong [33]. Qualitative research may also be useful to explore the reasons for the higher PA levels among Chinese children on weekends.

This study has several limitations. Higher response rate was sought from schools in districts with low SES compared with medium and high SES. As a result, half of the participants were from low SES areas. Furthermore, children who had parents with lower education levels were more likely to agree to wear the accelerometer. As a result, the findings should be generalised to other children with caution. There is inconsistent evidence for cross-sectional associations of SES with PA in children and adolescents, including in Hong Kong [29]. The longitudinal influences of SES on PA and ST need further investigation. There is no universal consensus on accelerometer cut points for determining PA. The MVPA minutes reported are therefore inevitably influenced by the cut points applied. The use of the relative lower cut-off counts may partly explain the higher prevalence of Chinese children meeting PA recommendations. However, the pattern for longitudinal changes will not be affected. To make weekday–weekend comparisons, 3 valid days of accelerometer data, including 1 weekend day, were required, which reduced the sample size. Compliance in wearing an accelerometer has been found to be more difficult on weekends than on weekdays. In the current study, over 80 % of the sample monitored provided valid accelerometer data on at least 3 weekdays at each assessment wave; however, only 60 % of them met the weekend wearing-time criteria. This compliance rate is similar or even higher than that reported in previous studies [9, 17]. Retention strategies, specific for weekends, warrant further research. Although using one weekend day is a commonly applied inclusive criterion for accelerometer data [8, 9], it may allow for atypical weekend activity events to influence the results. However, the proportion of children who provided valid data for only one weekend day was less than 30 % at each time point; thus may not have significant influence on the results. Finally, comparisons with other studies using shorter epoch for the accelerometer should be cautious since the epoch may have impact on the estimated PA levels for children [34].

## Conclusions

Chinese children were more physically active on weekends than weekdays. An age-related decline in MVPA was more marked on weekends than weekdays. Interventions to hinder age-related decline in PA and increase in ST should target both time segments, but weekends warrant particular attention for interventions targeting PA maintenance due to the greater declines of PA.

## Abbreviations

BMI: Body mass index
PA: Physical activity
MVPA: Moderate-to-vigorous physical activity
LPA: Light-intensity physical activity
ST: Sedentary time
UCAN: Understanding Children’s Activity and Nutrition
